# Evaluation of Dried Blood Spots with a Multiplex Assay for Measuring Recent HIV-1 Infection

**DOI:** 10.1371/journal.pone.0107153

**Published:** 2014-09-18

**Authors:** Kelly A. Curtis, Krystin M. Ambrose, M. Susan Kennedy, S. Michele Owen

**Affiliations:** Laboratory Branch, Division of HIV/AIDS Prevention, National Center for HIV/AIDS, Hepatitis, STD, and TB Prevention, Centers for Disease Control and Prevention, Atlanta, Georgia, United States of America; Temple University School of Medicine, United States of America

## Abstract

Laboratory-based HIV tests for recent infection (TRIs), which primarily measure a specific serological biomarker(s) that distinguishes recent from long-term HIV infection, have facilitated the estimation of population-based incidence. Dried blood spots (DBS) on filter paper are an attractive sample source for HIV surveillance, given the simplified and cost-effective methods of specimen collection, storage, and shipment. Here, we evaluated the use of DBS in conjunction with an in-house multiplex TRI, the HIV-1-specific Bio-Plex assay, which measures direct antibody binding and avidity to multiple HIV-1 analytes. The assay performance was comparable between matched plasma and DBS samples from HIV-1 infected individuals obtained from diverse sources. The coefficients of variation, comparing the median antibody reactivity for each analyte between plasma and DBS, ranged from 2.78% to 9.40% and the correlation coefficients between the two sample types ranged from 0.89 to 0.97, depending on the analyte. The correlation in antibody reactivity between laboratory and site-prepared DBS for each analyte ranged from 0.87 to 0.98 and from 0.90 to 0.97 between site-prepared DBS and plasma. The correlation in assay measures between plasma and DBS indicate that the sample types can be used interchangeably with the Bio-Plex format, without negatively impacting the misclassification rate of the assay.

## Introduction

Since the first AIDS cases were identified more than 30 years ago [1], significant advances have been made towards diagnosing HIV infection, developing anti-retroviral therapy (ART) regimens, and implementing transmission prevention measures. Despite this ever-evolving progress, an HIV vaccine remains elusive and an estimated 34 million people globally are living with HIV infection [1]. HIV surveillance methods have been instrumental in monitoring the status of the epidemic; however, surveillance has primarily focused on the prevalence of HIV in the population. Estimates of HIV incidence are crucial for understanding the dynamics of the epidemic and assessing the efficacy of prevention measures [2]; however, recent acquisition of HIV infection is difficult to extrapolate from standard diagnostic test results.

In 1998, a ground-breaking study by Janssen *et al.* described the development of a detuned or less-sensitive serologic assay for distinguishing recent from long-term HIV-1 infection, which allowed for incidence estimation from cross-sectional patient samples. Several serology-based laboratory tests for recent infection (TRI) have been developed that measure a specific biomarker, primarily HIV-1-specific antibody [3], [4], avidity [5]–[9], or both [10], [11], that evolves in a predictable pattern from early to late infection. To date, two TRIs have been commercialized for HIV-1 surveillance purposes, the BED-CEIA (Sedia Biosciences Corp., Portland, OR; Calypte Biomedical Corp., Portland, OR) and HIV-1 Limiting Antigen (LAg)-Avidity EIA (Sedia Biosciences Corp.; Maxim Biomedical, Inc., Rockville, MD). The BED assay has been used to calculate HIV-1 incidence estimates in the United States and worldwide. [12]–[15].

Given that the majority of current TRI approaches are serology-based, the most commonly used sample type for incidence testing is plasma and/or serum. Although HIV-1-specific antibodies are stable in plasma and serum, sample collection is somewhat limited due to processing and storage requirements. Dried blood spots (DBS) have been used extensively for HIV testing and surveillance, as they can be prepared from a finger stick, do not require centrifugation, and can be shipped at room temperature. HIV biomarkers remain stable on the DBS filter paper; with minimal risk of infectivity once the sample is fully dried [16]. Applications for DBS are numerous, including HIV-1-specific antibody testing, genotyping, viral nucleic acid amplification [17], [18], and drug resistance testing [19]. Furthermore, the use of DBS as a sample source for HIV-1 incidence estimation has been reported [20]–[22]. Protocol adaptations for DBS are currently supplied by the manufacturer for use with both the BED (Sedia Biosciences Corp.; Calypte Biomedical Corp.) and LAg assays (Maxim Biomedical Inc.). Collection of whole blood on DBS filter paper increases the applicability of laboratory-based TRIs, given that sample collection is suitable for multiple settings, including resource limited sites where the epidemic is often concentrated.

To improve upon the accuracy of TRIs for estimating incidence, it has been demonstrated that multiassay algorithms (MAAs), combining one or more TRIs with clinical data, yield improved incidence estimates and reduced false-recent rates as compared to each individual test [23]–[26]. Recently, we described the development of an in-house HIV-specific multiplex assay, based on the Bio-Plex format, which measures HIV-specific antibody levels and avidity to multiple analytes [10]. Improved incidence estimates have been demonstrated using multi-analyte algorithms based on three or more assay measures obtained from the multiplex format [27]. Thus far, all assay development for the HIV-1-specific Bio-Plex assay has been performed with plasma samples. In the current study, we evaluate the use of DBS as an additional sample source for determining recent infection with the multiplex assay. We compared assay performance with matched plasma and DBS samples from HIV-1 infected individuals obtained from diverse sources.

## Materials and Methods

### Study cohorts

Longitudinal seroconversion panels were obtained through the Seroconversion Incidence Panel Project (SIPP) in collaboration with SeraCare Life Sciences, Inc. (Milford, MA). The objective of the study was to identify recent HIV-1 seroconverters, as defined as having acquired HIV within the past 90 days. Donors were enrolled at plasma donation centers and HIV testing and/or clinical sites within the United States. Study participants were at least 18 years of age and met either of the following criteria: (1) detectable viral RNA with a negative or indeterminate antibody test result or (2) positive HIV antibody test with a documented negative test result within the past 90 days. Based on these criteria, 20 HIV-1 seroconverters were identified and enrolled for follow-up blood collection. Whole blood collection in EDTA vacutainers was initiated within 120 days from the date of the last negative or indeterminate HIV antibody test result. Blood samples were collected monthly for the first six months, followed by every three months for up to 15 months. Follow-up blood collections were continued after 15 months for most study participants (total follow-up time ranged from 189 to 1130 days). Twelve of the study participants had documented antiretroviral (ARV) use during the sample collection period. Cross-sectional samples from HIV-1 infected individuals with chronic or long-term infection were also collected. The last 2–3 samples collected from each of the seroconverters were included in the current study, along with 6 of the cross-sectional, long-term specimens (49 specimens/26 participants).

Cross-sectional samples from HIV-1 infected men who have sex with men (MSM) were obtained through CDC's National Behavioral and Surveillance (NHBS) system, as part of the MSM3 cycle. HIV-1 infected MSM were identified through routine testing in five US cities (Baltimore, Denver, Los Angeles, Miami, and Washington DC), as described in detail [28]. Briefly, whole blood in EDTA was collected from individuals with an initial HIV-1 rapid test result or self-reported HIV positives. All participants with a preliminary positive test result were confirmed HIV positive through site-specific HIV-1 diagnostic testing algorithms. Last negative and first positive HIV-1 antibody test results are not available for these participants. For the purposes of this study, 51 specimens were randomly selected from the NHBS cohort for inclusion in the analyses. All SIPP and NHBS specimens are from individuals with subtype B HIV-1 infections.

Lastly, cross-sectional specimens were obtained from HIV-1 infected donors from Cameroonian blood banks, as described elsewhere [19], [29]. Whole blood was collected as part of a surveillance study to characterize global HIV subtype diversity. A total of 52 Cameroonian specimens were included in the current study and determined to be from diverse, non-B subtype HIV-1 infections, as described [19], [29]. Recent/long-term HIV-1 infection status is unknown for the Cameroonian cohort, since last negative and first positive test results are not available for these participants. All samples were unlinked from personal identifiers and determined not to be human subjects research by the Centers for Disease Control and Prevention.

### Sample preparation

For the SIPP and NHBS cohorts, whole blood was shipped to the CDC at ambient temperature, within 48 hours of collection. DBS were made from whole blood by aliquoting 50 µl blood per spot on Whatman 903 Protein Saver cards (GE Healthcare Life Sciences, Piscataway, NJ). Each card was dried overnight at room temperature and packaged individually in Bitran bags (Fisher Scientific Company, Pittsburgh, PA) with silica gel desiccant bags and humidity indicating cards (McMaster-Carr Supply Co, Atlanta, GA). The DBS were stored at -20°C until ready for use. Matched plasma samples were prepared from the whole blood and stored at −80°C prior to testing. In addition to the CDC-prepared specimens, DBS were also prepared at the time of collection at one of the NHBS testing sites. Of the 51 NHBS participants included in this study, 14 had matched CDC and site-prepared DBS.

Sample collection and storage for the Cameroonian samples have been described previously [19], [29].

### DBS elution

A standard paper punch was used to obtain a 6.3 mm (diameter) blood spot from the DBS cards. Each blood spot was transferred to one well in a flat-bottom 96-well plate with 250 µl pre-incubation buffer (PBS with 1% BSA, 0.5% polyvinylalcohol (Sigma-Aldrich, St. Louis, MO), and 0.8% polyvinylpyrrolidone (Sigma-Aldrich)) [10] per well. The plate containing the blood spots was incubated overnight at room temperature to allow sample elution. The eluted sample was added directly into the Bio-Plex assay plate (50 µl sample/well).

### HIV-specific Bio-Plex assay

The HIV-1-specific Bio-Plex assay was performed on the matched plasma and DBS samples, as described [10], [27]. Magnetic COOH beads (Bio-Rad Laboratories, Hercules, CA) were coupled to the recombinant HIV-1 proteins, gp120, gp160, and gp41 (Immunodiagnostics, Inc., Woburn, MA). The recombinant proteins were derived from subtype B strains HIV-1 IIIB (gp120 and gp160) and MN (gp41). All plasma/eluted DBS samples were tested in duplicate under both treatment conditions, with and without diethylamine (DEA), and normalized mean fluorescent intensity (MFI) values and avidity indices were calculated as previously described [10], [27]. The normalized MFI value for gp41 was not included in the study analyses given that the analyte does not elicit sufficient separation in assay reactivity between recent and long-term specimens [10] and, therefore, is not useful for estimating HIV-1 incidence. A representative incident and prevalent sample was included in each run to assess inter-run variation. The standards were obtained from an HIV-1 Incidence/Prevalence performance panel (SeraCare Life Sciences, Milford, MA).

### Data analysis

To establish a baseline level of variation associated with the assay platform, intra-assay variation was assessed by calculating the coefficient of variation (CV) for the MFI values of each sample replicate. CVs were also calculated for the incident/prevalent standards included in each run. To evaluate variation associated with sample type, the CV comparing HIV-specific IgG antibody reactivity of plasma versus DBS was calculated for each analyte. The relationship between antibody reactivity of the plasma and DBS was also determined using Spearman's rank correlation coefficient.

### Determination of recent infection

To determine whether sample type affects the ability of the Bio-Plex assay to discriminate between recent and long-term HIV infection, cutoff values were determined for each analyte. In the absence of finalized cutoff values for the overall assay, the range of reactivity for known recent and long-term samples was determined using an HIV-1 Incidence/Prevalence Performance Panel (SeraCare Life Sciences). Given the minimal to no overlap between the reactivity of the recent and long-term panel members, the top end of the 95% confidence interval for the reactivity of the recent samples was selected as a cutoff for the purposes of the current analyses. For the study specimens, recent infection was defined as any value below the analyte-specific cutoff.

## Results

### Comparison of Plasma and DBS

**Figure 1:**
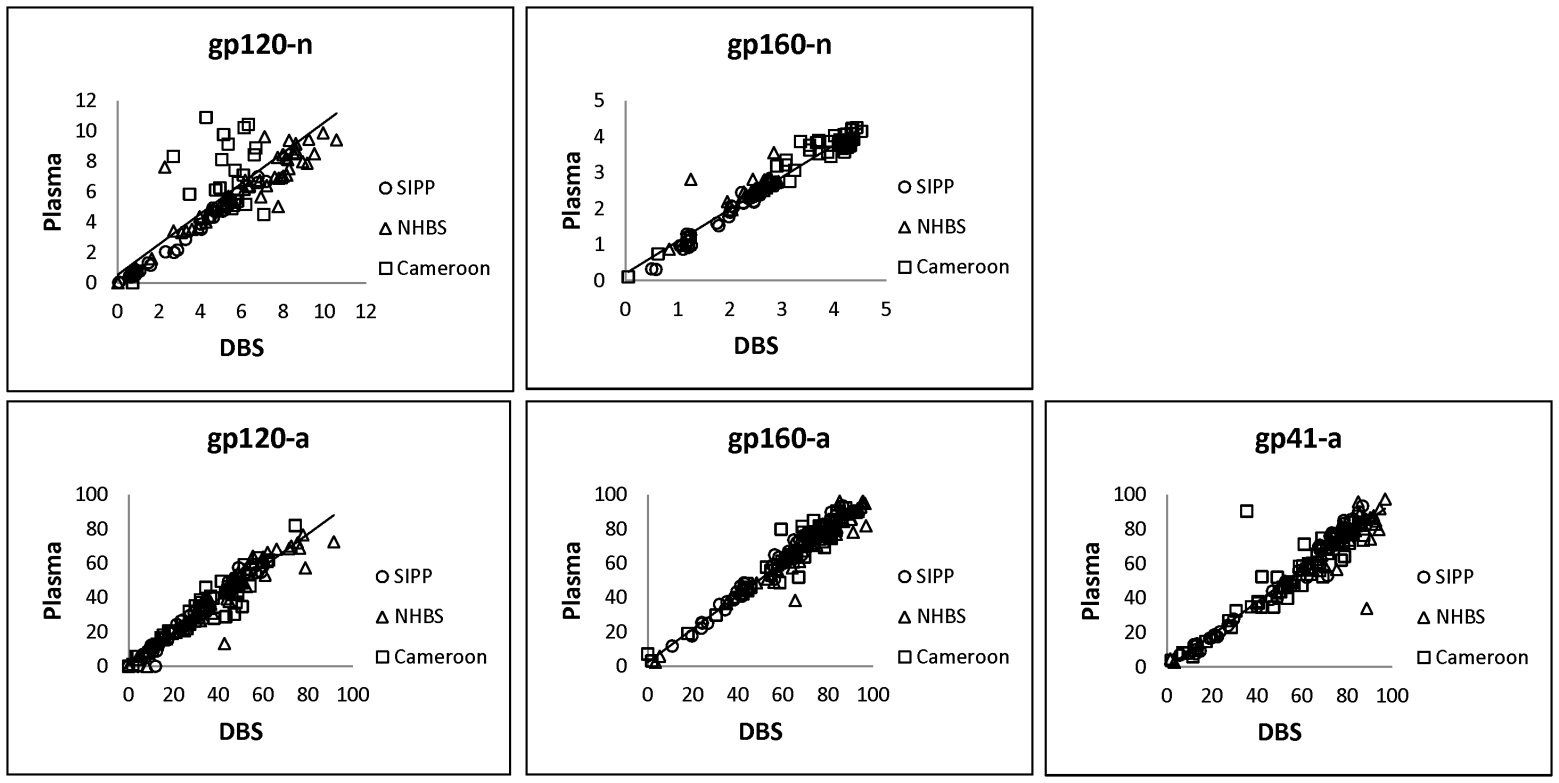
Correlation between plasma and DBS. The normalized MFI values (n) and avidity index (a) for plasma versus DBS were compared for all specimens. The solid line represents the linear trend line for all data points.

Intra-assay reproducibility was relatively high for both plasma and DBS samples for all analytes (Table 1). The median coefficient of variation (CV) of the sample replicates for each analyte ranged from 0.84 to 1.96 for plasma and 0.66 to 1.49 for DBS. A comparison of the normalized MFI values (n) and avidity index (a) for both sample types is shown in Figure 1. The variation in antibody reactivity between plasma and DBS was similar to the intra-assay variation, as measured by the CVs of the standards included in each assay plate (Table 2). The correlation coefficient (*r*) between assay values for plasma and DBS ranged from 0.89 to 0.97 for all cohorts. When analyzed separately, the avidity measures exhibited highly reproducible results for all cohorts (*r*≥0.9), while the correlation in normalized values was slightly more variable between cohorts (*r*≥0.8).

**Table 1:** Intra-assay variability.

Analyte	Intra-assay CV (%)^a^	
	Plasma	DBS
gp120	1.96	1.49
gp160	0.84	0.66
gp41	0.85	0.72

a Calculated based on MFI values of intra-assay sample replicates.

**Table 2:** Correlation in antibody reactivity between plasma and DBS.

Analyte	CV%		Correlation Coefficient (*r*)			
	Inter-assay standards^a^	Plasma vs. DBS^b^	SIPP (n = 49)	NHBS (n = 51)	Cameroon (n = 52)	All (n = 152)
**gp120-n**	11.14	9.40	1.00	0.90	0.83	0.89
**gp160-n**	6.71	3.81	0.99	0.79	0.95	0.97
**gp120-a**	7.00	4.28	0.99	0.96	0.94	0.96
**gp160-a**	7.33	2.78	0.99	0.97	0.96	0.97
**gp41-a**	3.99	5.09	0.99	0.93	0.89	0.95

a Inter-assay variation, as measured by repeat testing of know Incident/Prevalent controls. CV is representative of median values for standards.

b Median value for all samples, comparing plasma and DBS values.

### Comparison of site and CDC specimens

**Figure 2:**
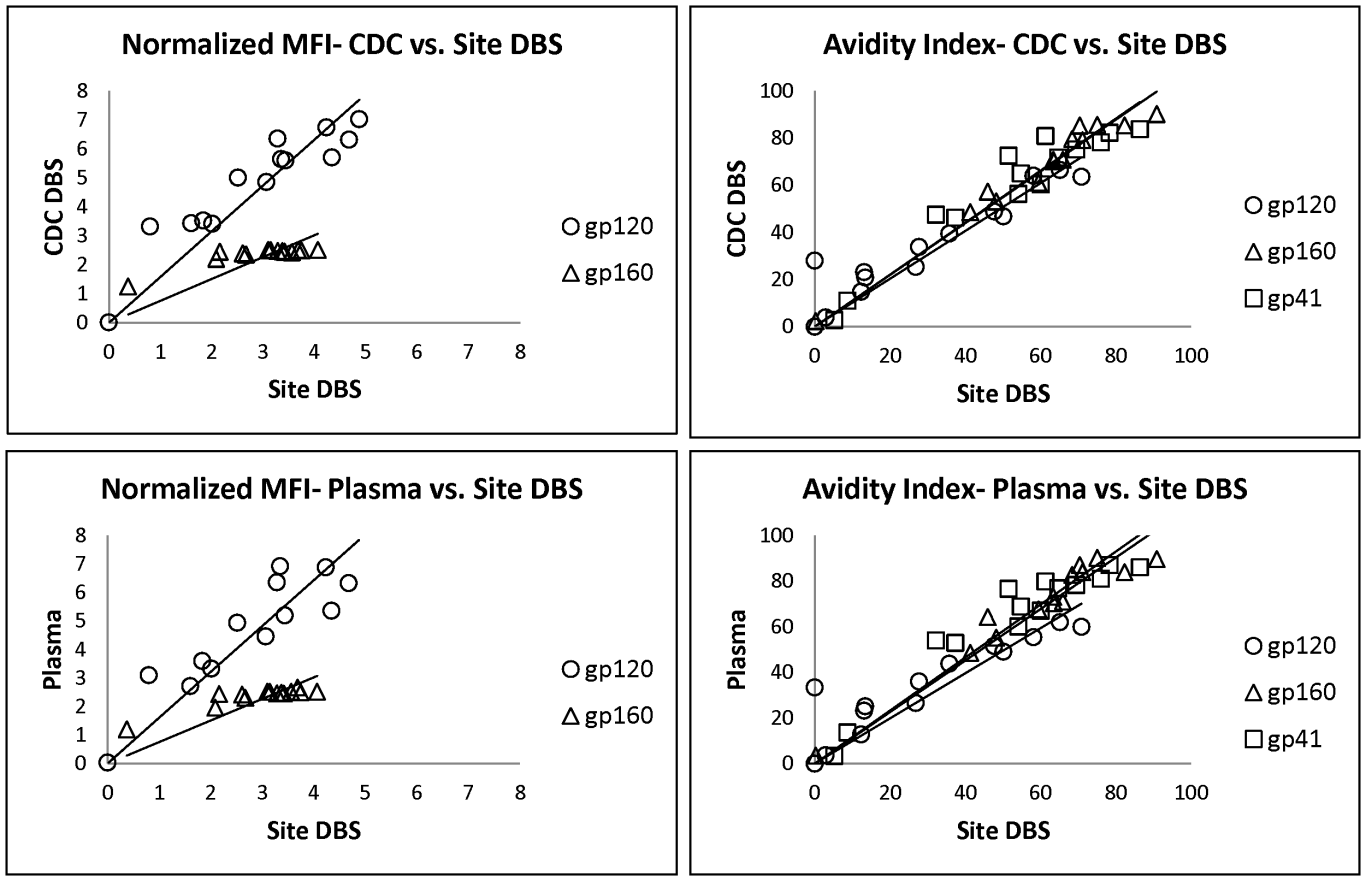
CDC versus field DBS. The normalized MFI values and avidity index were compared for CDC versus field-prepared DBS. The solid lines represent the linear trend lines.

The correlation in assay reactivity between CDC and site-prepared DBS was highly reproducible, with *r* values ranging from 0.87 to 0.98 for all analytes (Table 3). Similarly, the site-prepared DBS yielded assay values consistent with the plasma specimens (*r* = 0.90 to 0.97). A comparison of the normalized MFI values and avidity index for the CDC versus site-prepared specimens is shown in Figure 2.

**Table 3:** Correlation with site-prepared DBS.

Analyte	Correlation Coefficient (*r*)	
	CDC DBS (n = 14)	Plasma (n = 14)
gp120-n	0.93	0.91
gp160-n	0.87	0.90
gp120-a	0.94	0.91
gp160-a	0.98	0.97
gp41-a	0.95	0.95

### Determination of recent infection

Cutoff values for each analyte, chosen based on the assay reactivity of the performance panel, are listed in Table 4. For the study cohorts, all specimens with assay values below the analyte-specific cutoff were considered recent. The numbers of recent infections for plasma and DBS samples are shown in Table 4. For most analytes, the number of specimens determined to be recent were similar for both sample types. For plasma and DBS, the number of recent infections were identical for gp120-a and gp160-a. Recent infections differed by one for gp120-n and two for gp41-a. The largest discrepancy was for gp160-n, in which the recent infections differed by five samples.

**Table 4:** Number of recent infections.

Analyte	Cutoff Value	# Recent^a^	
		Plasma	DBS
gp120-n	2.3	29	30
gp160-n	2.5	56	61
gp120-a	11.6	26	26
gp160-a	26.8	11	11
gp41-a	24.1	24	26

a Number of samples classified as recent out of 152 total specimens.

## Discussion

In previous studies, we described the development and performance characteristics of an in-house HIV-1-specific Bio-Plex assay for determination of recent HIV-1 infection with plasma specimens [10], [27], [30]. Here, we evaluate the use of DBS eluates as an additional sample source for the customized Bio-Plex assay. The detection of HIV-1 antibodies from DBS using the Luminex technology (Luminex Corporation, Austin, TX) has been demonstrated for diagnostic purposes [31]; however, tests for recent infection require unique considerations given that the time of infection must be estimated with a fair degree of accuracy. Furthermore, our assay approach incorporates measurement of both total HIV-1-specific antibody binding and avidity; therefore, it is crucial to demonstrate that neither measure is significantly altered by sample type. Overall, comparable performance was demonstrated between matched plasma and DBS samples for both direct antibody binding and avidity measures. The consistency between sample types suggests that plasma and DBS can be used interchangeably with the Bio-Plex format, without negatively impacting the misclassification rate of the assay.

Given that most assays are subject to some degree of innate variation, it is important to characterize the baseline intra- and inter-assay variation prior to assessing potential differences in sample types. Specimen replicates yielded highly reproducible assay measures, irrespective of sample type. These findings were consistent with a previous study demonstrating performance of our in-house HIV-1 Bio-Plex assay versus a prototype kit, using plasma specimens [30]. Although samples were plated in duplicate for the purposes of the assay evaluation, the high degree of reproducibility supports the use of a single well for specimen testing, which improves the cost effectiveness of the assay. For all samples tested, the assay measures were remarkably similar between plasma and DBS for most analytes and any differences between the sample types could not be discerned from the innate variation associated with assay.

Specimens from three separate cohorts were evaluated in this study to assess potential inconsistencies due to site-specific sample collection or storage methods. Not surprisingly, the avidity measures were the most consistent between the cohorts. As observed in previous studies, avidity measures tend to be less affected by slight alterations in the assay or sample, given that avidity index is calculated relative to a control included within the same assay plate [10], [27], [30]. The correlation in normalized values between plasma and DBS tended to vary slightly between the cohorts. The SIPP cohort yielded highly consistent results for all analytes, with *r* values of 0.99 or 1. This may be the result of well-controlled sample and data collection, along with the fact that the specimens included in the current study were collected from a single site and freeze-thaw cycles were minimal for the plasma specimens. The NHBS cohort also exhibited high correlation between sample types; however, a lower *r* value of 0.79 for gp160-n was skewed by a single outlier. Unfortunately, limited sample volume for this particular study participant prevented repeat testing. For this particular cohort, site-prepared DBS were also available for some of the study participants. We did not observe any differences in reactivity between the site and CDC-prepared samples, which demonstrates that samples can be collected at field sites, shipped, and stored for later testing. The Cameroon specimens were the oldest of the three cohorts, as they were collected and stored 10–15 years ago and the DBS had been freeze-thawed multiple times for previous testing. Additionally, the Cameroon cohort exhibited a large degree of HIV-1 subtype diversity. Despite the age of the specimens and subtype diversity, the correlation between sample types was high for all analytes. It is important to note that any differences in reactivity between sample types might be exaggerated given the small sample numbers included in current study.

One of the most important considerations for a TRI is whether the recent/long-term classification is impacted by the use of DBS, thereby affecting incidence estimates. Since “true” recent/long-term status is not known for all of the samples included in this study, we compared the number of recent infections as determined by the assay values for plasma versus DBS. This evaluation was done strictly for demonstrative purposes, as finalized cutoffs have yet to be determined for the assay. Although demonstrated with a relatively small sample set, the number of specimens classified as recent were similar (differences ≤2) between plasma and DBS for all analytes except gp160-n. The number of specimens classified as recent differed between the analytes, given that each analyte has a distinct mean duration of recency (MDR) and, therefore, the determination of recent infection is analyte-specific [27]. Differences in classification between sample types were associated with specimens that elicited assay values close to the analyte cutoff. It is likely that misclassifications due to values close to the cutoff will decrease when the analyte measures are used in an algorithm to determine recent infection. The largest difference in recent classifications was observed for gp160-n, which is not surprising given the narrow range of reactivity between recent and long-term specimen. Given the high MFI values elicited from the gp160 bead set, titration of the gp160 protein on the beads can be performed to improve the measurable distinction between recent and long-term specimens (data not shown). Although each analyte was evaluated separately in the current study, an algorithm of analyte measures employing a common cutoff and MDR will be used to determine recent infection, as described previously [27]. The optimal algorithms for the current assay will be addressed in a separate study.

Given that most TRI formats are laboratory-based, the use of DBS as an additional sample source is highly valuable since samples can be collected in resource-limited settings and shipped for later testing. We demonstrate stable reactivity of antibodies from DBS, even after specimens have been stored for several years. The modification to utilize DBS for the HIV-1 Bio-Plex assay described here expands its utility as a TRI for estimating HIV incidence.
